# ThWRKY4 from *Tamarix hispida* Can Form Homodimers and Heterodimers and Is Involved in Abiotic Stress Responses

**DOI:** 10.3390/ijms161126009

**Published:** 2015-11-13

**Authors:** Liuqiang Wang, Lei Zheng, Chunrui Zhang, Yucheng Wang, Mengzhu Lu, Caiqiu Gao

**Affiliations:** 1 State Key Laboratory of Tree Genetics and Breeding, Key Laboratory of Tree Breeding and Cultivation of the State Forestry Administration, Research Institute of Forestry, Chinese Academy of Forestry, Beijing 100091, China; liuqiangwang2009@yahoo.com; 2 State Key Laboratory of Tree Genetics and Breeding (Northeast Forestry University), 26 Hexing Road, Harbin 150040, China; zhenglei8123@126.com (L.Z.); zcr_sherry@163.com (C.Z.); wangyucheng@ms.xjb.ac.cn (Y.W.)

**Keywords:** WRKY, stress response, dimerization, *Tamarix hispida*, yeast two-hybrid

## Abstract

WRKY proteins are a large family of transcription factors that are involved in diverse developmental processes and abiotic stress responses in plants. However, our knowledge of the regulatory mechanisms of WRKYs participation in protein–protein interactions is still fragmentary, and such protein–protein interactions are fundamental in understanding biological networks and the functions of proteins. In this study, we report that a WRKY protein from *Tamarix hispida*, ThWRKY4, can form both homodimers and heterodimers with ThWRKY2 and ThWRKY3. In addition, ThWRKY2 and ThWRKY3 can both bind to W-box motif with binding affinities similar to that of ThWRKY4. Further, the expression patterns of *ThWRKY2* and *ThWRKY3* are similar to that of *ThWRKY4* when plants are exposed to abscisic acid (ABA). Subcellular localization shows that these three ThWRKY proteins are nuclear proteins. Taken together, these results demonstrate that ThWRKY4 is a dimeric protein that can form functional homodimers or heterodimers that are involved in abiotic stress responses.

## 1. Introduction

WRKY proteins compose a large family of plant transcription factors (TFs) and play pivotal roles in defence signalling as well as regulating diverse growth and developmental processes [1]. The proteins feature at least one highly conserved WRKY domain, which is composed of an approximately 60 amino acid stretch with the sequence WRKYGQK at the N-terminus and a zinc finger structure at the C-terminus [2,3]. WRKY proteins can be grouped into three different classes according to the number of WRKY domains and the structure of the zinc finger motif [2]. More than half of the members are classified into group I, which are proteins that contain two WRKY domains and a C_2_H_2_ zinc-finger motif. The members of Group II feature a single WRKY domain and a C_2_H_2_ zinc-finger motif, and are further sub-classified into IIa+b, IIc and IId+e, whereas proteins with a single WRKY domain and a C_2_–H–C zinc-finger-like motif compose group III [4]. Numerous studies have demonstrated that WRKY proteins specifically bind to the W-box motif, which has the core sequence “TTGACC/T” in the promoters of target genes [5,6,7,8].

Recently, the functionally characterized WRKY proteins from several plant species have been found to play important roles in various aspects of plant developmental processes, including leaf senescence [9,10], flowering [11,12], trichome formation [13], and seed development [14,15]. Moreover, members of the WRKY family also participate in defense responses to biotic pathogens [16,17] as well as abiotic stress responses to environmental stimuli and hormones [18,19,20]. WRKY proteins also play important roles in certain plant hormone signal transduction pathways, such as abscisic acid [21,22], jasmonic acid [10], and salicylic acid [23]. Despite their biological functional diversity, almost all analyzed WRKY proteins recognize the W-box sequence and physically interact with a wide range of proteins, e.g., WRKY, VQ (FxxxVQxLTG) motif proteins, MIOTGEN-ACTIVATED PROTEIN KINASE (MAPK), chromatin remodeling proteins, and calmodulin [24,25]. For example, the AtWRKY18, AtWRKY40, and AtWRKY60 interact with themselves, like AtWRKY6 and AtWRKY42, and they also interact with each other via a leucine zipper motif at the *N*-terminus [26,27,28]. The *Arabidopsis* Group III protein AtWRKY30 prominently interacts with AtWRKY53, AtWRKY54, and AtWRKY70 and forms heterodimers with members of group IIb [29]. In addition, the 34 *Arabidopsis* VQ motif-containing proteins can interact with WRKY proteins in yeast [30]. In rice, OsWRKY33 was reported to interact with OsBWMK1 using yeast two-hybrid screening. *In vitro* assays have shown that OsWRKY33 is phosphorylated by OsBWMK1, and results in enhancing its DNA binding activity [31]. Another study demonstrated that OsWRKY30 can interact with other rice MAPKs and can be phosphorylated [32].

Previously, we reported a working model for the function of *ThWRKY4* from *Tamarix hispida* in abiotic stress responses [8]; mechanisms in addition to simple recognition of the W-box element are necessary for the regulatory specificity of the ThWRKY4 protein. In the present study, we further demonstrated that ThWRKY4 can form homodimers or heterodimers using the yeast two-hybrid system, and we investigated whether the partners of ThWRKY4 can bind to the W-box motif using yeast one-hybrid and transient expression assays. Overall, the study provides helpful insights into the functions of WRKY and defines the roles of WRKY in abiotic stress responses in plants.

## 2. Results and Discussion

### 2.1. Analysis of the Hetero- and Homo-Dimers of ThWRKY4

Our previous study showed that ThWRKY4 could bind to the W-box element and regulate the targeted genes involved in abiotic stress response, thereby conferring abiotic stress tolerance to transgenic *Arabidopsis* plants [8]. In this study, we studied whether ThWRKY4 could homodimerize or heterodimerize using a yeast two-hybrid (Y2H) system. Twelve unique WRKYs with full coding sequences (CDSs) were identified from the transcriptomes of *T. hispida*, and these *WRKY* genes were designated *ThWRKY1* to *ThWRKY12*. We performed multiple sequence alignments for these proteins with four homologous WRKYs from *Arabidopsis* (Figure S1A). A phylogenetic tree showed that these ThWRKYs and other plant stress-responsive WRKYs form three main subgroups, which suggests that they belong to different subgroups (Figure S1B).

**Figure 1:**
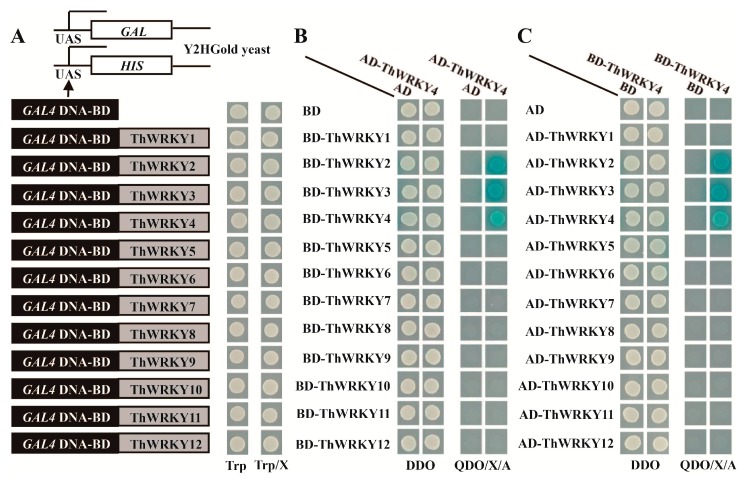
Yeast two-hybrid (Y2H) analysis of the dimerization of ThWRKY4. (**A**) Diagram of the ThWRKYs that were fused to the *GAL4* DNA binding domain in the yeast pGBKT7 vector. The transformed yeast culture was dropped onto SD/–Trp plates with X-α-Gal (Trp/X) to investigate transcriptional activation; (**B**,**C**) Protein interactions in yeast two-hybrid systems. Twelve ThWRKY proteins were individually fused to the *GAL4* DNA-AD domain in pGADT7 following the above manner. The Y2HGold yeast cells were co-transformed with the indicated plasmids and dropped onto SD/–Leu/–Trp (DDO) and SD/–Ade/–His/–Leu/–Trp/X-α-Gal/Aureobasidin A (QDO/X/A) plates to examine growth at 30 °C for 3–4 days.

Subsequently, these 12 CDSs of *ThWRKY*s were individually cloned into pGBKT7 to investigate their transcriptional activation. We found that the transformed yeast cells (Y2HGold) grew well on SD/–Trp (Figure 1A), but could not grow on the SD/–Ade/–His/–Trp medium. These results show that the 12 ThWRKYs do not show transcriptional activation, and are therefore suitable for the Y2H assay. These ThWRKYs (including ThWRKY4) were individually fused in frame to the *GAL4* activation domain in pGADT7 vector, and the interactions between ThWRKY4 and other ThWRKYs or itself were studied to investigate the homodimerization and heterodimerization of ThWRKY4. Y2H assays were performed using ThWRKY4 in pGBKT7 with ThWRKY4 and other ThWRKYs in pGADT7. As shown in Figure 1B,C, the Y2H results show that ThWRKY4 can interact with itself and can bind to ThWRKY2 and ThWRKY3. To further confirm these results, ThWRKY4 harboring in pGADT7 was interacted with the ThWRKYs harboring in pGBKT7 using the Y2H assay. Consistently, the results show that ThWRKY4 can bind to itself, ThWRKY2 and ThWRKY3. These results indicate that ThWRKY4 can form homodimers with itself as well as heterodimers with ThWRKY2 or ThWRKY3.

Chi *et al.* [25] reported that WRKY proteins interact with a variety of proteins to either activate or repress transcription; therefore, it is important to study the partners of WRKYs to reveal their functions. Recently, increasing evidences demonstrate that WRKYs could form homodimers and heterodimers [24,27,29]. The dimerization of transcription factors is important for modifying binding-site specificities, which alters dimer stability.

### 2.2. Analysis of the Binding of ThWRKY2 and ThWRKY3 to the W-Box Motif

**Figure 2:**
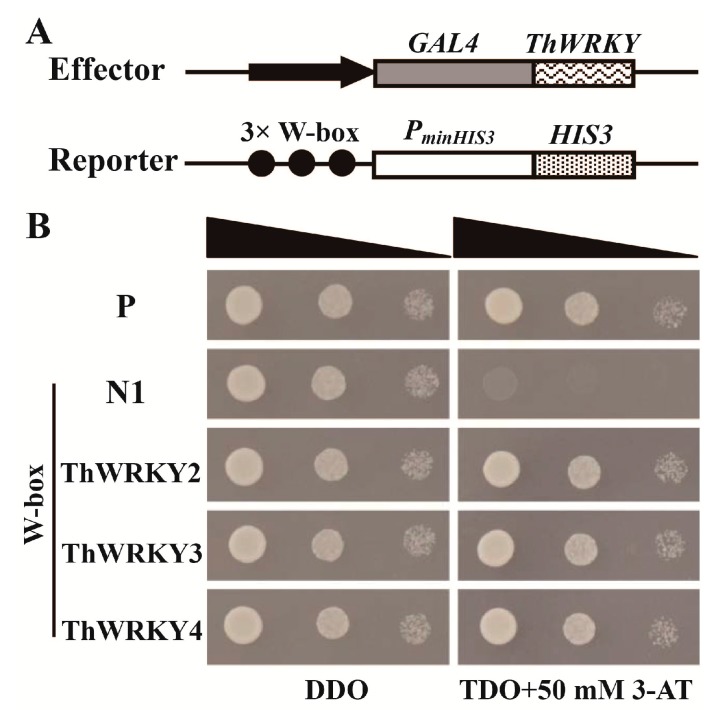
Analysis of the binding of ThWRKY2, ThWRKY3 and ThWRKY4 to the W-box motif in yeast. (**A**) Diagram of the reporter and effector vectors. Three tandem copies of the W-box were inserted into the pHIS2 vector as the reporter construct. The CDSs of *ThWRKY*s were cloned into pGADT7-Rec2 as the effector constructs; (**B**) The effector and reporter constructs were co-transformed into the yeast strain Y187. Positive transformants were further identified by spotting serial dilutions (1:1, 1:10 and 1:100) of yeast onto SD/–Leu/–Trp (DDO) and SD/–His/–Leu/–Trp (TDO) plates with 3-AT. P: positive control (p53HIS2 + pGAD-Rec2-53); N: negative controls (pHIS2-W-box + pGAD-Rec2-53).

WRKY proteins specifically bind to the W-box motif with the core sequence “TTGACC/T” [6,19,20]. To determine whether ThWRKY2 and ThWRKY3 can bind to the W-box motif, as is the case for ThWRKY4, a yeast two-hybrid (Y1H) assay was performed. As shown in Figure 2B, all the yeast transformants grew well, and there were no differences in growth rates or clone sizes on the SD/–His/–Leu/–Trp (TDO) medium containing 50 mM 3-AT (3-amino-1,2,4-triazole), which suggests that, like ThWRKY4, both ThWRKY2 and ThWRKY3 can bind to the W-box motif.

**Figure 3:**
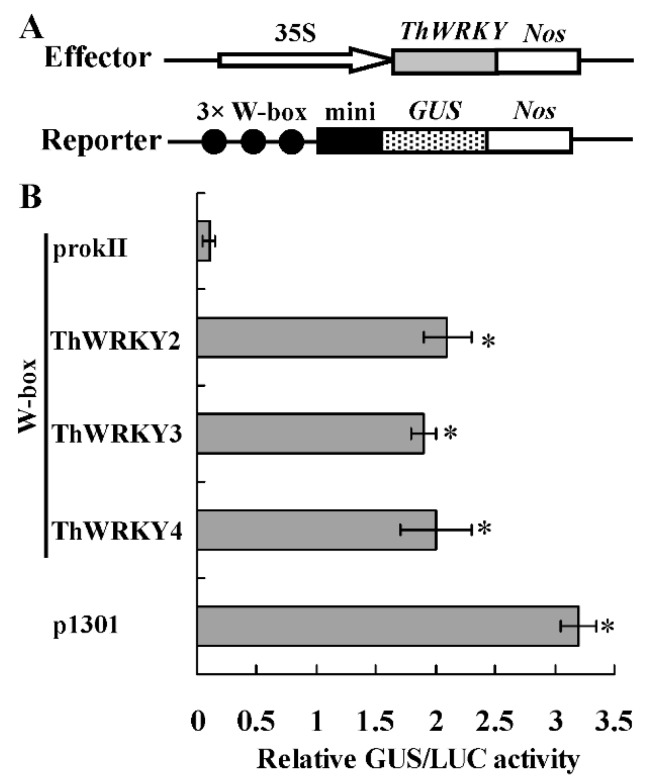
Analysis of the binding of ThWRKY2, ThWRKY3, and ThWRKY4 to the W-box motif in plants. (**A**) Diagram of the reporters and effectors. Triple tandem copies of the W-box were fused with the 35S CaMV −46 minimal promoter and cloned into pCAMBIA1301 for driving the *GUS* gene as the reporter construct. The CDSs of *ThWRKY*s into pROKII under the control of the 35S promoter as the effector constructs; (**B**) GUS activity assay of the co-expression of effector and reporter plasmids in tobacco leaves. Each effector and the reporter constructs were co-transformed into tobacco leaves. The 35S-luciferase construct was transformed together with the reporter and effector into leaves to normalize for transformation efficiency. The error bars were standard deviations, which were calculated from three independent biological repeats. * indicates a significant difference (*p* < 0.05).

To further confirm the results of the Y1H assay, the three tandem repeats of the W-box motif were inserted into the pCAMBIA1301 vector as the reporter, and the CDSs of *ThWRKY2*, *ThWRKY3*, *ThWRKY4* were cloned into pROKII vector as the effector. Each effector and a reporter were co-transformed into tobacco leaves by the particle bombardment method (Figure 3B). The transformation of ThWRKY2, ThWRKY3 or ThWRKY4 results in high levels of GUS activity, which suggests that each protein can interact with the W-box motif. Taken together, these results show that the ThWRKYs can specifically bind to the W-box motif and might regulate the expression of targeted genes containing the W-box motif in their promoter regions. Additionally, the binding affinities of the W-box motif to these three ThWRKYs are similar.

### 2.3. Expression of ThWRKY2, 3, and 4 in Response to Abiotic Stresses

**Figure 4:**
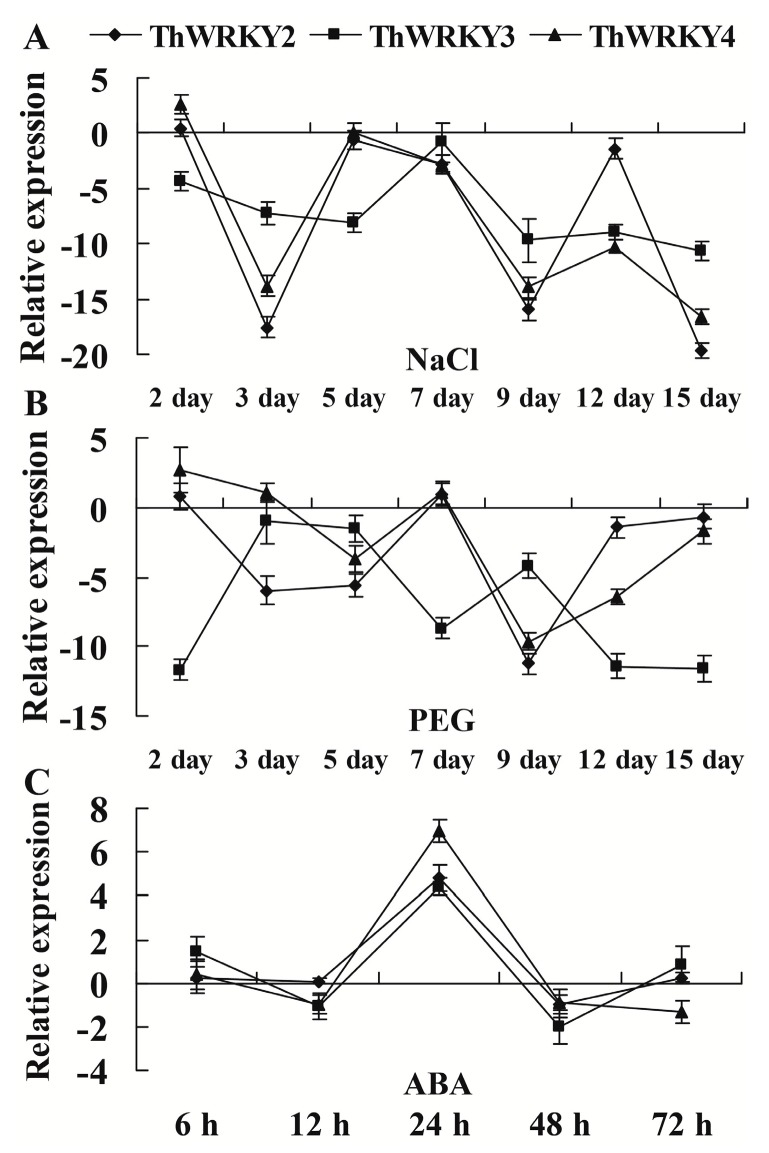
Expression profiles of *ThWRKY2*, *ThWRKY3* and *ThWRKY4* under different abiotic stresses. Uniformly developed 6-month-old *T. hispida* seedlings were treated with 300 mM NaCl (**A**), 15% (*w*/*v*) PEG6000 (**B**) or 100 μM ABA (**C**) for the indicated times. A fresh water-only control was conducted in parallel. After these treatments, the roots of seedlings from each sample were harvested and pooled for real-time RT-PCR analyses. The error bars were standard deviations, which were calculated from multiple replicates of real-time PCR.

To investigate the expression patterns of *ThWRKY2*, *ThWRKY3* and *ThWRKY4* in response to salt, drought and ABA treatments, a real-time RT-PCR analysis was performed (Figure 4). The expression levels of *ThWRKY2* and *ThWRKY4* share similar patterns under salt (300 mM NaCl) and PEG-induced drought (15% PEG6000) stress conditions. Their expression levels initially increased after two days of salt and PEG-induced drought stresses and then rapidly decreased at three days. At subsequent time points, they showed both increase and decrease, but their expression was completely suppressed during salt and PEG-simulated drought stresses. *ThWRKY3* expression was always downregulated during salt and PEG-induced drought stress. Zheng *et al.* [8] reported that the expression of *ThWRKY4* in the roots and leaves of 2-month-old *T. hispida* was upregulated or downregulated under salt (400 mM NaCl) and PEG-induced drought (20% PEG6000) stresses during a short time frame (3, 6, 9, 12, and 24 h). The differences in the *ThWRKY4* expression patterns may be caused by the different stress time points and stress concentrations. Interestingly, *ThWRKY2*, *ThWRKY3* and *ThWRKY4* shared very similar expression patterns in response to ABA stimulus. Their expression was slightly upregulated after 6 h of ABA treatment, gradually decreased at 12 h, markedly increased to the highest level at 24 h, and gradually reduced to relative low levels at subsequent time points. These results suggested that these genes may be involved in an ABA-dependent stress-signaling pathway.

### 2.4. Subcellular Localization of ThWRKY2, ThWRKY3, and ThWRKY4

**Figure 5:**
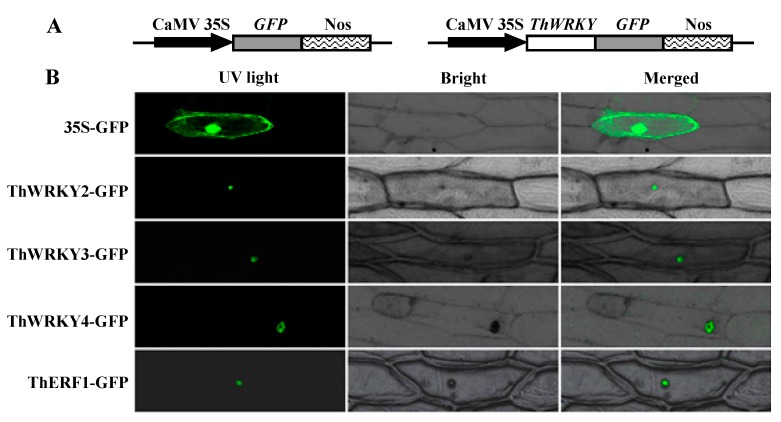
Nuclear localization of ThWRKY proteins. (**A**) Diagram of the 35S-GFP control construct and recombinant plasmid pBI121-ThWRKY-GFP; (**B**) The *ThWRKY-GFP* fusion gene and *GFP* (control) were transiently expressed in onion epidermal cells by the particle bombardment (Bio-Rad, Hercules, CA, USA) method. The transformed cells were cultured on MS medium for 24–36 h and visualized using a confocal microscope at 488 nm (LSM410, Zeiss, Jena, Germany). An ethylene-responsive factor from *T*. *hispida*, ThERF1, is a nuclear protein according to Wang *et al.* [37], which was used as a positive control.

To study the subcellular localization of ThWRKYs, the *ThWRKY-GFP* fusion gene and the *GFP* gene were respectively transformed into onion epidermal cells by the particle bombardment method. The control GFP was observed to be distributed throughout the transformed cells, whereas the ThWRKY-GFP fusion proteins were exclusively localized to the nucleus (Figure 5B), which suggests that these ThWRKY proteins are nuclear proteins. Many studies indicated that most plant WRKYs are present in the nucleus, such as BhWRKY1, PtrWRKY73, and AtWRKY42 [6,33,34], but a number of membrane-bound transcription factors are stored in their dormant forms in the cytoplasm and entered the nucleus only when activated by various environmental stimuli or hormones [35,36]. Our studies showed that these three ThWRKY proteins are localized in the nucleus even without being activated.

## 3. Experimental Section

### 3.1. Plant Materials and Growth Conditions

Seedlings of *Tamarix hispida* were planted in pots containing a mixture of turf peat and sand (2:1 *v*/*v*) under controlled greenhouse conditions of 70%–75% relative humidity, 14 h light, 10 h dark and an average temperature of 24 °C. Uniformly developed 6-month-old seedlings were exposed to 300 mM NaCl, 15% (*w*/*v*) PEG6000 for 2, 3, 5, 7, 9, 12, and 15 days or 100 μM ABA for 6, 12, 24, 48, and 72 h. A fresh water-only control was conducted in parallel. After each stress treatment, the roots of twenty seedlings were harvested and stored at −80 °C.

### 3.2. Cloning the ThWRKY Gene Sequences

Seven transcriptomes were built from the roots and leaves of *Tamarix hispida* treated with NaHCO_3_ by RNA-seq using high-throughput Solexa sequencing technology. A total of 94,359 non-redundant unigenes were generated using TGI clustering tools [36]. After functional annotation, 12 unigenes containing WRKY domains with full coding sequences (CDSs) were predicted in the protein database of NR. Multiple sequence alignments of these protein sequences together with homologous WRKYs from *Arabidopsis* were performed with ClustalW using BioEdit software and adjusted manually. The un-rooted phylogenetic tree was constructed with MEGA5.05 using the Neighbor-Joining (NJ) method, and the bootstrap values were estimated with 1000 replicates at each node [38].

### 3.3. Construction of the Yeast Expression Vector and Yeast Two-Hybrid Analysis

The CDSs of *ThWRKY*s were amplified and cloned into the *GAL4* DNA binding domain of the pGBKT7 vector, which served as the BD construct (Table S1). These *ThWRKY*s were cloned into the pGADT7-Rec vector as the AD constructs using the same methods. The BD constructs and empty pGBKT7 plasmid (control) were respectively transformed into the yeast strain Y2HGold and grown on SD/–Trp/X-α-Gal medium to test for transcriptional activation, because it is imperative to confirm that the bait does not autonomously activate the *AbAr* reporter gene in Y2HGold cell in the absence of an AD protein. To investigate the interactions of ThWRKY4 with itself and other ThWRKY proteins in the yeast two-hybrid assay, the recombinant BD and AD vectors were co-transformed into the yeast Y2HGold cells, which were then grown on SD/–Ade/–His/–Leu/–Trp/X-α-Gal/Aureobasidin A medium (QDO/X/A).

### 3.4. Yeast One-Hybrid Assays

To evaluate the binding of the ThWRKY proteins to the W-box (TTGACC), three tandem copies of the W-box were inserted into the pHIS2 multiple cloning sites (*Eco*RI and *Sac*I) the upstream of the *HIS3* reporter gene as the reporter construct, and the CDSs of the *ThWRKY* gengs were individually cloned into the pGADT7-Rec2 vector as effectors (Figure 2A, Table S2). Their interactions were studied using the yeast one-hybrid (Y1H) system (Clontech, Palo Alto, CA, USA). The p53HIS2 vector containing three copies of DNA motifs recognized by p53 and the pGADT7-Rec2-53 vector encoding murine p53 fused with the *GAL4* AD domain were respectively used as negative and positive control vectors.

### 3.5. Transient Expression Assays

To further study whether the ThWRKY proteins could bind to the W-box motif, triple tandem repeats of the W-box motif were fused with the minimal 35S CaMV promoter (−46 to +1) and replaced CaMV 35S promoter for driving the *GUS* gene in pCAMBIA1301 vector, this construct served as a reporter. The CDSs of *ThWRKY2*, *ThWRKY3*, and *ThWRKY4* were cloned into pROKII driven by the CaMV 35S promoter to generate effector constructs (Figure 3A, Table S2). The reporter and each effector vector were co-transformed into tobacco leaves by the particle bombardment method. At the same time, the CaMV 35S-luciferase (35S-LUC) construct was also transformed together to normalize for transformation efficiency. The GUS activity was determined according to the method of Jefferson *et al.* [39]. Each experiment was carried out by three independent biological repetitions.

### 3.6. Real-Time RT-PCR Analyses

Total RNA was isolated form each sample by the CTAB (hexadecyltrimethylammonium bromide) method [40]. First-strand cDNA synthesis and real-time PCR were performed according to Wang *et al.* [36]. The average values of the cycle thresholds (*C*_t_) of the α*-tubulin*, β*-tubulin*, and β*-actin* genes were used as internal references. The details of the primers used are listed in Table S3. Each experiment was carried out by three technical and three biological replicates. The relative expression ratios calculated from the cycle threshold (*C*_t_) according to the delta-delta *C*_t_ method [41]. In another words, the relative transcription level was calculated as the transcription level under stress treatment divided by the transcription level under control conditions.

### 3.7. Subcellular Localization Analysis

The CDSs of *ThWRKY*s (without the stop codon) were fused to the N-terminus of the green fluorescent protein (GFP) of pBI121 vector driven by the CaMV 35S promoter (Figure 5A, Table S4). The *35S-ThWRKY*s*-GFP* fusion gene and *35S-GFP* (control) were transiently expressed in onion epidermal cells by the particle bombardment (Bio-Rad) method. Then, the transformed cells were cultured on MS medium for 24–36 h and analyzed using a confocal laser scanning microscope at 488 nm (LSM410, Zeiss, Jena, Germany).

### 3.8. Statistical Analysis

Unless otherwise specified, each experiment was carried out by three independent biological replicates.

## 4. Conclusions

Previously, we reported the function of *ThWRKY4* involves in abiotic stress tolerance. Based on those results, we have further revealed that the ThWRKY4 protein can homodimerize with itself and heterodimerize with the ThWRKY2 and ThWRKY3 proteins, both of which bind to the W-box motif with similar binding affinities. In addition, *ThWRKY2* and *ThWRKY3* exhibited expression patterns similar to that of *ThWRKY4* in the roots of *T*. *hispida* under ABA treatment. These ThWRKYs were all targeted to the nucleus. As ThWRKY4 is tolerant of salt and drought stresses, the heterodimers of ThWRKY4 may also be involved in abiotic stress tolerance. This study provides useful information in revealing further details of the functions of ThWRKY4.

## Supplementary Materials

Supplementary materials can be found at http://www.mdpi.com/1422-0067/16/11/26009/s1.
