# Supplementary material for: ThWRKY4 from Tamarix hispida Can Form Homodimers and Heterodimers and Is Involved in Abiotic Stress Responses
Source: Int J Mol Sci. 2015 Nov 13;16(11):27097–106. doi: 10.3390/ijms161126009 (PMC4661867; doi:10.3390/ijms161126009)
Supplement: Supplementary file 1 [file ijms-16-26009-s001.pdf]

Supplementary Materials: ThWRKY4 from *Tamarix hispida* Can Form Homodimers and Heterodimers and Is Involved in Abiotic Stress Responses

Liuqiang Wang [1,†], Lei Zheng [2,†], Chunrui Zhang [2], Yucheng Wang [2], Mengzhu Lu [1,*] and Caiqiu Gao [2,*]

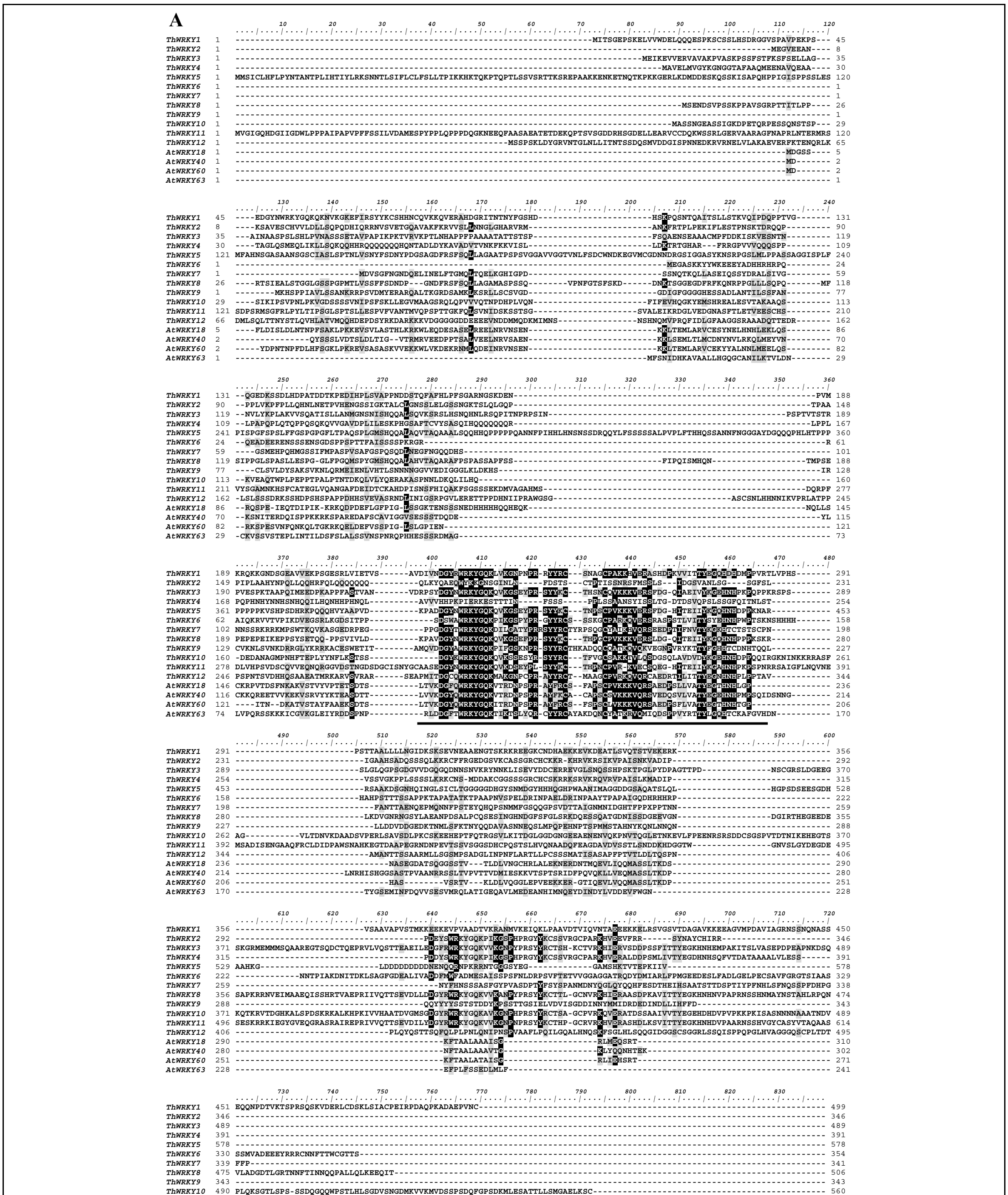

Figure S1. *Cont.*

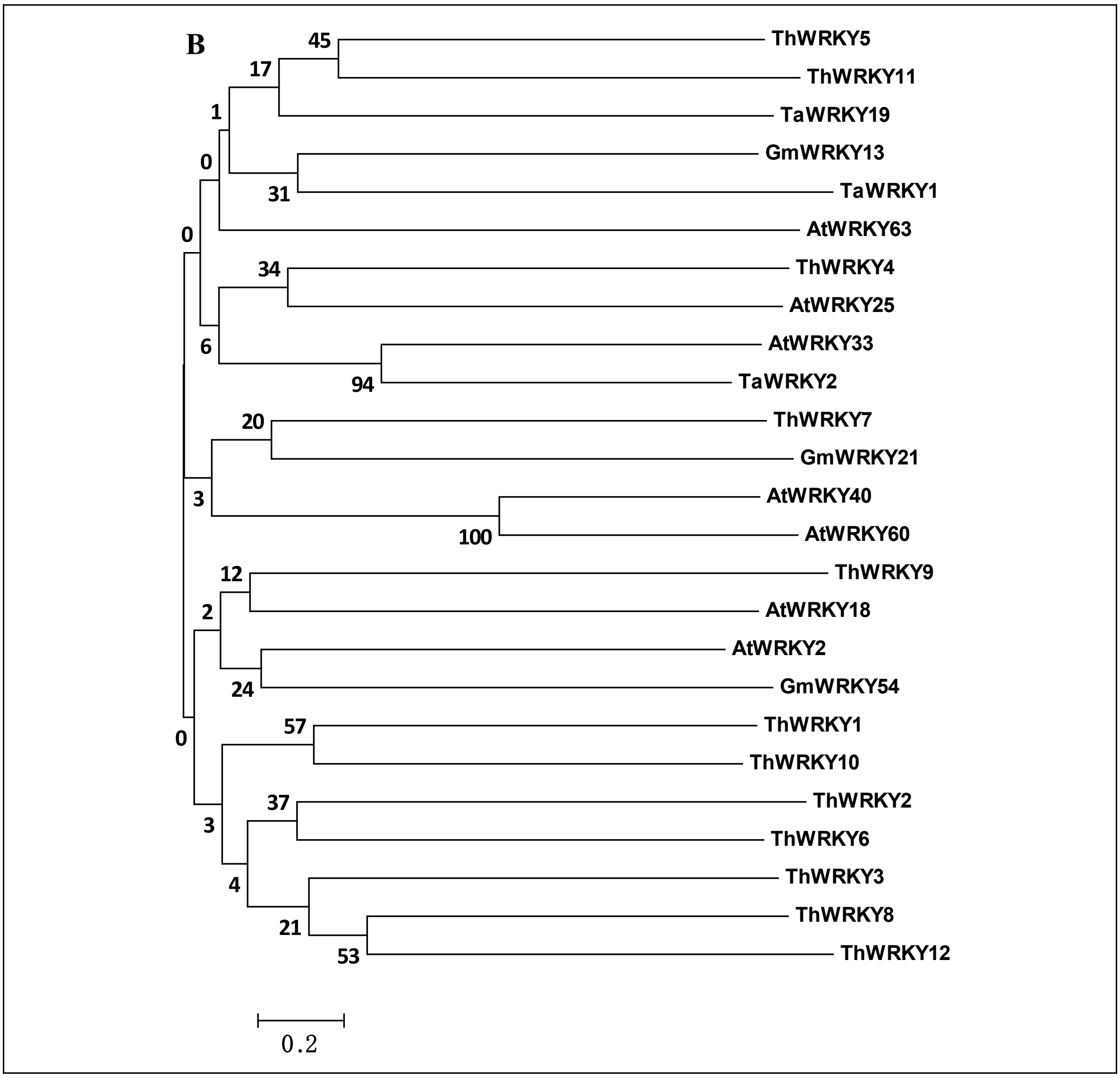

Figure S1. Sequence analysis of ThWRKYs. (**A**) Multiple sequence alignment of amino acid sequences of ThWRKYs with homologous WRKYs from *Arabidopsis* were performed with ClustalW using BioEdit software. The typical WRKY domain is underlined; The different boxes show the similar and identical of the sequences; (**B**) Phylogenetic tree of ThWRKYs and other plant stress-responsive WRKYs, which was constructed using MEGA 5.05 with 1000 bootstrap replicates.

Table S1. Primers used in constructing yeast expression vector.

Genes	GenBank Number	Construct	Forward and Reverse Primers (5′–3′)	
ThWRKY1	JX416191	AD-ThWRKY1	TGGCCATTATGGCCCGGGATGATTACTTCAGGGGAACC	GACATGTTTTTTCCCGGGCTAGCAGTTAACAGGTTC
		BD-ThWRKY1	CATGGAGGCCGAATTCATGATTACTTCAGGGGAACC	GCAGGTCGACGGATCCCTAGCAGTTAACAGGTTC
ThWRKY2	JX416192	AD-ThWRKY2	TGGCCATTATGGCCCGGGATGGAGGGGGTTGAGGAGGCT	GACATGTTTTTTCCCGGGTCACCTTCGTATGTGACAATAAG
		BD-ThWRKY2	CATGGAGGCCGAATTCATGGAGGGGGTTGAGGAGGCT	GCAGGTCGACGGATCCTCACCTTCGTATGTGACAATAAG
ThWRKY3	JQ040808	AD-ThWRKY3	TGGCCATTATGGCCCGGGATGGAGATTAAAGAGGTGGTG	GACATGTTTTTTCCCGGGTTACTGGCTATCTTTGTTAGG
		BD-ThWRKY3	CATGGAGGCCGAATTCATGGAGATTAAAGAGGTGGTG	GCAGGTCGACGGATCCTTACTGGCTATCTTTGTTAGG
ThWRKY4	JX416193	AD-ThWRKY4	TGGCCATTATGGCCCGGGATGGCAGTGGAATTAATGGTAG	GACATGTTTTTTCCCGGGCTACGATGATTCAAGCACG
		BD-ThWRKY4	CATGGAGGCCGAATTCATGGCAGTGGAATTAATGGTAG	GCAGGTCGACGGATCCCTACGATGATTCAAGCACG
ThWRKY5	JX416194	AD-ThWRKY5	TGGCCATTATGGCCCGGGATGTCAATCTGTCTTCATTTTCTTCC	GACATGTTTTTTCCCGGGAACTATGATCTTGGGTTCAGTCAC
		BD-ThWRKY5	CATGGAGGCCGAATTCATGTCAATCTGTCTTCATTTTCTTCC	GCAGGTCGACGGATCCAACTATGATCTTGGGTTCAGTCAC
ThWRKY6	JX416195	AD-ThWRKY6	TGGCCATTATGGCCCGGGATGGAAGGTGCAAGCAAGAAG	GACATGTTTTTTCCCGGGTCAACTAGTGGTCCCACACCAAG
		BD-ThWRKY6	CATGGAGGCCGAATTCATGGAAGGTGCAAGCAAGAAG	GCAGGTCGACGGATCCTCAACTAGTGGTCCCACACCAAG
ThWRKY7	JX416196	AD-ThWRKY7	TGGCCATTATGGCCCGGGATGGATGTCAGCGGTTTCAATG	GACATGTTTTTTCCCGGGTCACGGGAAAAATCCTGGG
		BD-ThWRKY7	CATGGAGGCCGAATTCATGGATGTCAGCGGTTTCAATG	GCAGGTCGACGGATCCTCACGGGAAAAATCCTGGG
ThWRKY8	JX416197	AD-ThWRKY8	TGGCCATTATGGCCCGGGATGTCCGAAAACGACTCTGTTC	GACATGTTTTTTCCCGGGCTACGTGATTTGTTCTTC
		BD-ThWRKY8	CATGGAGGCCGAATTCATGTCCGAAAACGACTCTGTTC	GCAGGTCGACGGATCCCTACGTGATTTGTTCTTC
ThWRKY9	JX416198	AD-ThWRKY9	TGGCCATTATGGCCCGGGATGAAGCACTCGCCGCCTATC	GACATGTTTTTTCCCGGGCTAATCAAAAAAATGAATCAGC
		BD-ThWRKY9	CATGGAGGCCGAATTCATGAAGCACTCGCCGCCTATC	GCAGGTCGACGGATCCCTAATCAAAAAAATGAATCAGC
ThWRKY10	JX416199	AD-ThWRKY10	TGGCCATTATGGCCCGGGATGGCTTCATCAAACGGAG	GACATGTTTTTTCCCGGGTCAGCAAGACTTGAGTTCTGC
		BD-ThWRKY10	CATGGAGGCCGAATTCATGGCTTCATCAAACGGAG	GCAGGTCGACGGATCCTCAGCAAGACTTGAGTTCTGC
ThWRKY11	JX416200	AD-ThWRKY11	TGGCCATTATGGCCCGGGATGGTTGGGATTGGTCAAC	GACATGTTTTTTCCCGGGTTATAAACAGATAACAGATG
		BD-ThWRKY11	CATGGAGGCCGAATTCATGGTTGGGATTGGTCAAC	GCAGGTCGACGGATCCTTATAAACAGATAACAGATG
ThWRKY12	JX416201	AD-ThWRKY12	TGGCCATTATGGCCCGGGATGTCCTCTCCTAGCAAATTG	GACATGTTTTTTCCCGGGTTAATAGCAGCTGCTAGACC
		BD-ThWRKY12	CATGGAGGCCGAATTCATGTCCTCTCCTAGCAAATTG	GCAGGTCGACGGATCCTTAATAGCAGCTGCTAGACC

The words in different colors indicate a homology to the linear ends (5′ and 3′) of pGADT7 and pGBKT7 vectors.

Table S2. Primer sequences employed in the yeast one-hybrid assay and transient expression assays.

Construct	Forward and Reverse Primers (5′–3′)	
pHIS2-W-box	AATTCTTGACCTTGACCTTGACCGAGCT	CGGTCAAGGTCAAGGTCAAG
pHIS2	GCCTTCGTTTATCTTGCCTGCTC	CGATCGGTGCGGGCCTCTTC
pGAD-ThWRKY2	TGGCCATTATGGCCCGGGATGGAGGGG GTTGAGGAGGCT	GACATGTTTTTTCCCGGGTCACCTT CGTATGTGACAATAAGC
pGAD-ThWRKY3	TGGCCATTATGGCCCGGGATGGAGATT AAAGAGGTGGTG	GACATGTTTTTTCCCGGGTTACTG GCTATCTTTGTTAGG
pGAD-ThWRKY4	TGGCCATTATGGCCCGGGATGGCAGTG GAATTAATGGTAG	GACATGTTTTTTCCCGGGCTACGA TGATTCAAGCACG
pGAD	CTATTCGATGATGAAGATACCCCACCA AACCC	GTGAACTTGCGGGGTTTTTCAGTA TCTACG
pROKII-ThWRKY2	CTCTAGAGGATCCCCGGGATGGAGGGG GTTGAGGAGGCT	TCGAGCTCGGTACCCGGGTCACCT TCGTATGTGACAATAAGC
pROKII-ThWRKY3	CTCTAGAGGATCCCCGGGATGGAGATT AAAGAGGTGGTG	TCGAGCTCGGTACCCGGGTTACTG GCTATCTTTGTTAGG
pROKII-ThWRKY4	CTCTAGAGGATCCCCGGGATGGCAGTG GAATTAATGGTAG	TCGAGCTCGGTACCCGGGCTACG ATGATTCAAGCACG
pROKII	TTTCATTTGGAGAGAACACG	TGCCAAATGTTTGAACGATC
pCAM-W-box	AGCTTTTGACCTTGACCTTGACCACCCT TCCTCTATATAAGGAAGTTCATTTCATT TGGAGAGAACACGGC	CATGGCCGTGTTCTCTCCAAATGA AATGAACTTCCTTATATAGAGGAA GGGTGGTCAAGGTCAAGGTCAAA
pCAMBIA1301	TAGAGTCGACCTGCAGGCAT	ATCATCATCATAGACACACG

The words in different colors and the underline indicate a homology to the linear ends (5′ and 3′) of pGADT7, pROKII or pCAMBIA1301 vectors and different sites of restriction endonucleases.

Table S3. Gene-specific primers used in real-time RT-PCR

Genes	GenBank Number	Forward and Reverse Primers (5′–3′)	
ThWRKY2	JX416192	CCACCACCATTAGTCAA	AATCCCGCTATTACCCT
ThWRKY3	JQ040808	CCCAAGTTAAGTCGCGATC	GAATGCGTGCATTTGTAGT
ThWRKY4	JX416193	CAGAATCACCACCCTCATAAT	TAGCATCGTCCATAGAGTTAC
β-actin	FJ618517	AAACAATGGCTGATGCTG	ACAATACCGTGCTCAATAGG
α-tubulin	FJ618518	CACCCACCGTTGTTCCAG	ACCGTCGTCATCTTCACC
β-tubulin	FJ618519	GGAAGCCATAGAAAGACC	CAACAAATGTGGGATGCT

Table S4. Primer sequences employed in subcellular localization analysis

Construct	Forward and Reverse Primers (5′–3′)	
pBI121-ThWRKY2-GFP	TCTAGACTGGTACCCGGGATG GAGGGGGTTGAGGAGGCT	CTAGTCAGTCGACCCGGGTCA CCTTCGTATGTGACAATAAG
pBI121-ThWRKY3-GFP	TCTAGACTGGTACCCGGGATG GAGATTAAAGAGGTGGTG	CTAGTCAGTCGACCCGGGTTA CTGGCTATCTTTGTTAGG
pBI121-ThWRKY4-GFP	TCTAGACTGGTACCCGGGATG GCAGTGGAATTAATGGTAG	CTAGTCAGTCGACCCGGGCTA CGATGATTCAAGCACG
pBI121-GFP	TTTCATTTGGAGAGAACACG	CGACCAGGATGGGCACCAC

The red words indicate a homology to the linear ends (5′ and 3′) of pBI121 vectors.
